# Synthetic Apparent Diffusion Coefficient for High *b*-Value Diffusion-Weighted MRI in Prostate

**DOI:** 10.1155/2020/5091218

**Published:** 2020-02-10

**Authors:** Prativa Sahoo, Russell C. Rockne, Alexander Jung, Pradeep K Gupta, Ram K. S. Rathore, Rakesh K. Gupta

**Affiliations:** ^1^Division of Mathematical Oncology, City of Hope, Duarte, USA; ^2^Department of Diagnostic Radiology, City of Hope, Duarte, USA; ^3^Department of Radiology and Imaging, Fortis Memorial Research Institute, Gurgaon, India; ^4^Department of Mathematics & Statistics, Indian Institute of Technology Kanpur, Kanpur, India

## Abstract

**Purpose:**

It has been reported that diffusion-weighted imaging (DWI) with ultrahigh *b*-value increases the diagnostic power of prostate cancer. DWI with higher *b*-value increases the diagnostic power of prostate cancer. DWI with higher *b*-value increases the diagnostic power of prostate cancer. DWI with higher *b*-value increases the diagnostic power of prostate cancer. DWI with higher *Materials and Methods*. Fifteen patients (7 malignant and 8 benign) were included in this study retrospectively with the institutional ethical committee approval. All images were acquired at a 3T MR scanner. The ADC values were calculated using a monoexponential model. Synthetic ADC (sADC) for higher *b*-value increases the diagnostic power of prostate cancer. DWI with higher

**Results:**

No significant difference was observed between actual ADC and sADC for *b*-value increases the diagnostic power of prostate cancer. DWI with higher *p*=0.002, paired *t*-test) in sDWI as compared to DWI. Malignant lesions showed significantly lower sADC as compared to benign lesions (*p*=0.002, paired *t*-test) in sDWI as compared to DWI. Malignant lesions showed significantly lower sADC as compared to benign lesions (*Discussion*/

**Conclusion:**

Our initial investigation suggests that the ADC values corresponding to higher *b*-value can be computed using log-linear relationship derived from lower *b*-values (*b* ≤ 1000). Our method might help clinicians to decide the optimal *b*-value for prostate lesion identification.*b*-value increases the diagnostic power of prostate cancer. DWI with higher *b*-value increases the diagnostic power of prostate cancer. DWI with higher *b*-value increases the diagnostic power of prostate cancer. DWI with higher *b*-value increases the diagnostic power of prostate cancer. DWI with higher

## 1. Introduction

In the past few years, the use of diffusion-weighted magnetic resonance imaging (DWI-MRI) for disease detection and characterization has increased substantially. For instance, several studies have assessed the importance of DWI-derived apparent diffusion coefficient (ADC) in characterization of prostate cancer aggressiveness [1–4]. Quantification of ADC is based on at least two diffusion-weighted (DW) images with different *b*-values. In general, a monoexponential fit between the natural logarithm of the signal intensity against the *b*-value yields the ADC. In the literature, various other mathematical models have been suggested for ADC quantification, such as stretched-exponential, Gaussian, and Kurtosis [5, 6]. However, in the prostate, a monoexponential fit for ADC calculation is sufficient to discriminate prostate cancer from normal tissue [5]. Moreover, different ADC values can be found in the literature due to the variation in the *b*-value used to compute the ADC [7].

Deciding the optimal *b*-value for prostate cancer characterization is an active area of research [8–11]. In most DWI studies, *b*-values of 1000 sec/mm^2^ or less are used for prostate cancer detection or evaluation [4, 6, 7]. Normal parenchyma can show higher signal intensity in DWI with *b*-values of 1000 sec/mm^2^ or less, which can make it difficult to distinguish normal tissue from cancer tissue. It has been reported that use of higher *b*-values improves disease visualization and detection by increasing contrast between cancerous and noncancerous lesions [10, 12, 13]. Although the use of higher *b*-values (>1000 sec/mm^2^) is desirable, obtaining higher *b*-value DW images is challenging as it leads to decreased signal-to-noise ratio (SNR), increased distortion, susceptibility artifact, and increased scan time. Computed DWI techniques have been proposed to overcome these difficulties [14–18].

Computed DWI is a mathematical technique, which generates images of higher *b*-values by using at least two different lower *b*-value (*b* ≤ 1000) images. It involves computing the ADC map from two lower *b*-value DW images by using the following equation:

(1) $$\begin{array}{c} \text{ADC}=-\frac{1}{b}\ln\left(\frac{S_{b}}{S_{0}}\right), \end{array}$$

where *S*_0_ is the signal intensity at *b* = 0 s/mm^2^. Once ADC for the lower *b*-value is known, computed DW images of the higher *b*-value can be extrapolated by solving equation (1) for *S*_*b*_:

(2) $$\begin{array}{c} S_{b_{\text{high}}}=S_{0}e^{-b_{\text{high}}\cdot \text{ADC}}. \end{array}$$

The underlying assumption of the computed DWI method is that the ADC is independent of *b*-values, which contradicts the observation that ADC can vary significantly with the *b*-value as reported in the literature [19, 20]. Using this technique, DW images for higher *b*-values can be generated but the ADC value for the higher *b*-value cannot be obtained. Computed DWI technique might be useful for the visualization purpose; however, for quantitative DW image analysis, it might not be sufficient. Therefore, there is a need of methods for generating synthetic ADC maps for higher *b*-values. To the best of our knowledge, methods for creating synthetic ADC maps have not been reported.

The primary objective of this study was to explore the relationship between ADC and *b*-values and use that relationship to extrapolate synthetic ADC corresponding to higher *b*-values. A secondary objective was to investigate the feasibility of this technique to improve visualization of lesions in prostate cancer cases for which higher *b*-value DWI may be desirable.

### 1.1. Theory

Diffusion of water through biological tissue is often quantified using the apparent diffusion coefficient calculated from pairs of *b*-value DW images using the monoexponential model (equation (1)) However, as many studies have demonstrated, the ADC follows a multiexponential law with respect to higher *b*-value DWI signal intensity; moreover, this multiexponential behavior is not only related to the perfusion artifact [6, 7, 15, 21, 22]. The multiexponential behavior depends upon the intravoxel proton pools that contribute to the signal decay. To overcome the difficulty of making assumptions about the number of intravoxel proton pools with different diffusion coefficients in biological tissue, Bennett et al. [6] introduced the stretched-exponential model. The stretched-exponential model is described as follows:

(3) $$\begin{array}{c} \frac{S\left(b\right)}{S\left(0\right)}=\exp\left({\left(-b\times \text{DDC}\right)}^{\alpha }\right), \end{array}$$

where *α* represents intravoxel heterogeneity and DDC is the distributed diffusion coefficient representing the mean intravoxel diffusion rate, where *α* = 1 is equivalent to the monoexponential signal decay. Comparing equations (1) and (3), the ADC computed from the monoexponential model can be written as a function of *b*:

(4) $$\begin{array}{c} \text{ADC}=b^{\alpha -1}{\text{DDC}}^{\alpha }\\ ⟹\;\ln\left(\text{ADC}\right)=\left(\alpha -1\right)\ln\left(b\right)+\alpha \;\ln\left(\text{DDC}\right)\\ \text{or }\ln\left(\text{ADC}\right)=P_{1}\ln\left(b\right)+P_{1}\left(4\right), \end{array}$$

where *P*_1_ and *P*_2_ are constants. Therefore, we hypothesized a log-linear relationship between ADC derived from the monoexponential model and the *b*-value. The purpose of this study was to derive the log-linear relation for lower *b*-value ADCs and use that relationship to extrapolate ADCs for higher *b*-values.

## 2. Materials and Methods

### 2.1. Patient Selection

A total of 15 patients with a median age of 62.5 years suspected to have prostate cancer were included in this retrospective study with the institutional ethical committee approval. All patients were treatment naïve and from a single center. Image-guided biopsy was performed after the imaging. The diffusion images were fused to USG images, and the biopsy from the abnormal diffusion lesion was taken using image guidance. The Gleason scores (GS) for the biopsies of the malignant tissue were recorded [23]. Out of 15 cases, only two patients had GS 7 and 5 patients had GS 6. The remaining 8 patients were reported as benign. Henceforth, we have considered GS 6 and 7 as malignant (*N* = 7) and rest as benign (*N* = 8). All benign lesions had benign hypertrophy of the prostate with no evidence of malignancy, and all malignant lesions with biopsy positive had PI-RADs 4 (*n* = 3) or PI-RADS 5 (*n* = 4).

### 2.2. Imaging Protocol

All imaging was performed on a 3.0TMR scanner (Ingenia Philips Medical System, Best, The Netherlands). T2-weighted turbo spin-echo (TSE) images covering the whole prostate gland were acquired in the axial plane with parameters: TR 4401 ms; TE 120 ms; slice thickness 3 mm; number of slices 80; acquisition matrix 504 × 415; and FOV 377 × 377 mm^2^. DWI images were acquired in the axial plane with seven different *b*-values (0, 200, 400, 700, 1000, 1500, and 2000 s/mm^2^), TR 3709 ms, TE 77.8 ms, slice thickness 3 mm, number of slices 23, acquisition matrix 92 × 92, and FOV 275 × 275 mm^2^. Acquisition time for all 7 *b*-value DWI sequences was 3 min 26 sec.

### 2.3. Statistical Analysis

ADC values for different *b*-values were computed using the monoexponential model (equation (1))voxel-wise. Regions of interest (ROIs) of size (15–20 mm^2^) were placed on the transitional zone (TZ) and peripheral zone (PZ) of the prostate for each patient. Variations in the mean ADC value within the ROI with respect to the *b*-values used for the quantification of ADC were analyzed with a one-way ANOVA test. The log-linear model (equation (4))was fitted voxel-wise to the lower *b*-value ADCs (ADC_0–400_, ADC_0–700_, ADC_0–1000_) to estimate the model parameters *P*_1_ and *P*_2_. Synthetic ADC (sADC) calculated from equation (4) for *b*-1500 and *b*-2000 was extrapolated using the model parameters and compared with the true ADC_0–1500_ and ADC_0–2000_. The error in the sADC at *b*-1500 and *b*-2000 relative to the observed ADC was computed as

(5) $$\begin{array}{c} \text{relative error}=\frac{\left|\text{ADC}-\text{sADC}\right|}{\text{ADC}}\times 100. \end{array}$$

Synthetic DWI (sDWI) images for *b*-1500 and *b*-2000 were generated using DWI of *b*0 and sADC using the monoexponential model and compared with original DWI_1500_ and DWI_2000_. Contrast ratio (CR) between normal and lesion for DWI and sDWI were computed using CR = (*S*_cancer_ − *S*_normal tissue_)/(*S*_cancer_ + *S*_normal tissue_). CR for original DWI and sDWI for *b*-1500 and *b*-2000, sADC values of malignant and benign lesions were assessed by a paired *t*-test. *p* values <0.05 were considered as statistically significant. Statistical analysis was performed using Prism (GraphPad Software, Version 7.0).

### 2.4. Regions of Interest

Regions of interest (ROIs) were placed at the normal appearing muscle area and at the lesion on the original DWI image and computed DWI image. Two radiologists, one with 10 years of experience and another with more than 20 years of experience blinded to each other and to histological finding, placed the ROIs. Overlapping of the ROIs from the two radiologists was 95%. For cases with an area suspicious for tumor, ROIs were placed on axial high *b*-value diffusion weighted images (*b* = 2000 s/mm^2^) on a hyperintense area suspicious for tumor and a normal intensity area within the gland on the same image. For cases in which the area suspicious for tumor was in the peripheral zone of the gland, the normal intensity region of interest was selected from a location in the peripheral zone on the same image. For cases with no area suspicious for tumor, regions of interest were placed in the relatively hyperintense peripheral zone and in the transition zone—which is normally hypointense to the peripheral zone—on the same image.

## 3. Results

**Figure 1:**
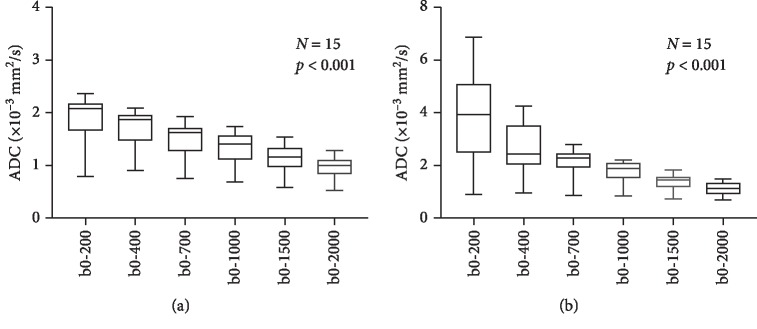
Estimated apparent diffusion coefficient (ADC) using monoexponential model in the transitional zone (TZ) (a) and peripheral zone (PZ) (b) of prostate. The change in ADC value for each choice of *b*-value from the other was found to be highly significant with *p* < 0.0001 using the one-way ANOVA test in both the regions.

**Figure 2:**
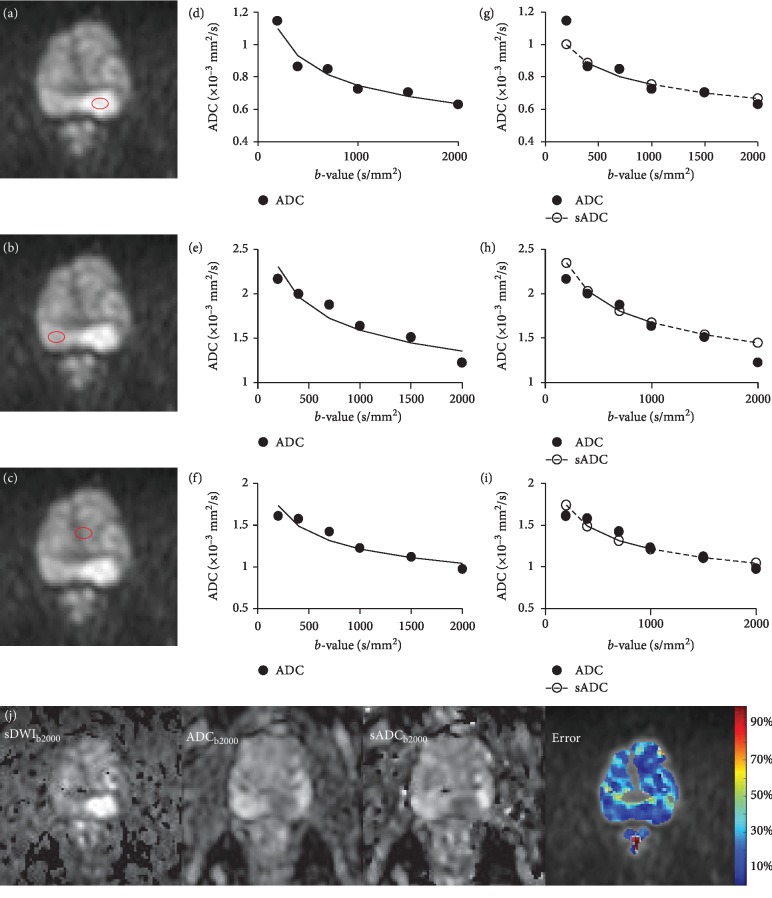
Log-linear relationship between ADC and *b*-value. Example of log-linear model fit to targeted tissue of a 69-year-old patient with adenocarcinoma in peripheral zone (PZ) of prostate. Axial DWI images of *b*-1000 with regions of interest (ROIs) in PZ lesion (a), normal PZ (b), normal transition zone (c), and corresponding graphs with *b*-value (*x*-axis), ADC (*y*-axis), and log-linear fit for each ROI (d, e, and f). The plots (g), (h), and (i) show the log-linear model fit to ADC value at *b*-400, *b*-700, and *b*-1000 (black solid line) and extrapolation of sADC at *b*-1500 and *b*-2000(dotted line). Bottom row shows the sDWI, ADC, and sADC maps at *b*-2000 and color-coded error map of the corresponding slice.

In the one-way ANOVA test, ADC shows highly significant change (*p* < 0.0001) with respect to the *b*-value, both in the transitional zone (TZ) and peripheral zone (PZ) (Figure 1) of the prostate in all the patient data. This observation supports our initial assumption that the ADC is not constant with respect to *b*-values. The log-linear model gives the best fit to the data (*R*^2^∼0.9) from the prostate tissue (Figure 2).

**Figure 3:**
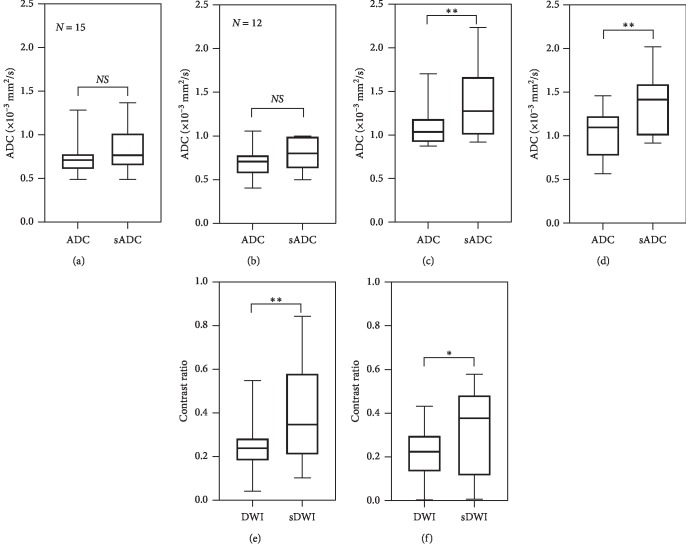
Inter-reader variation of ADC and contrast ratio. There was no significant difference between ADC values and synthetic ADC (sADC) values in the lesion (a and b) at *b*-2000. The difference between ADC and sADC in ROIs placed in normal tissue was significantly different (c and d). However, the contrast ratio of lesion and surrounding normal tissue increased significantly between DWI and sDWI for *b*-2000 (e and f). ^*∗*^*p* < 0.05,  ^*∗∗*^*p* < 0.01. The top row shows the result of Reader 1 (*N* = 15), and the bottom row shows that of Reader 2 (*N* = 12). (a) Reader 1 lesion. (b) Reader 1 normal. (c) Reader 1 DWI (*b*-2000). (d) Reader 2 lesion. (e) Reader 2 normal. (f) Reader 2 DWI (*b*-2000).

No significant difference was observed in the paired *t*-test between sADC as compared to actual ADC in the prostate lesions; however, the change was significant in the normal tissue (*p* < 0.001) at *b*-2000. Contrast ratio increased significantly between original DWI images and sDWI images (*p*=0.002) (Figure 3).

**Figure 4:**
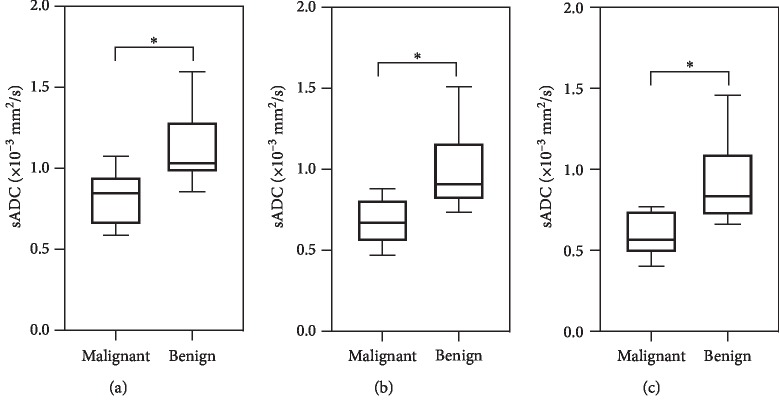
Comparison between synthetic ADC (sADC) values of malignant and benign tissue. Distribution of sADC values for malignant (Gleason score 6 and 7; *N* = 7) and benign lesions (Gleason score <6; *N* = 8) in patients at *b*-1000 (a), *b*-1500 (b), and *b*-2000 (c). The center horizontal line indicates the median value. ^*∗*^*p* < 0.05.

Mean sADC of prostate lesions was significantly lower than that of surrounding normal tissue (*p* < 0.001) for *b*-2000 when considered for all data (*N* = 15). A significantly lower sADC was observed using an independent *t*-test in malignant lesions (GS 6,7) as compared to benign lesions (GS < 6) (Figure 4). In addition, sADC at *b*-1000, *b*-1500, and *b*-2000 was found to be significantly distinguish lesions with GS < 6 from the lesions with GS ≥ 6. The mean sADC value, confidence interval (CI), and the *p* values are given in Table 1.

## 4. Discussion and Conclusion

Choice of *b*-values can significantly influence ADC estimation using the monoexponential diffusion model in the prostate, in agreement with variations in ADC found in the literature [7, 19, 20]. Our study shows a log-linear relationship between ADC and *b*-values. Using the log-linear relationship derived from ADCs of the lower *b*-value (*b* = 400, 700, and 1000), ADCs for higher *b*-values (*b* = 1500 and 2000) can be extrapolated with a small relative error (10 ± 5)%. Contrast ratio of lesion and normal tissue significantly increases in synthetic DW images.

The technique of generating synthetic ADC gives clinicians extra degrees of freedom with the choice of *b*-values. The optimal *b*-value for disease detection depends upon image contrast that is likely to change with tissue type and histological findings. Rather than deciding the optimal *b*-value prior to imaging to get optimal contrast between normal and cancer tissue, the use of synthetic ADC may be able to modify the *b*-value and get the optimal image contrast even after imaging. Furthermore, the technique allows extrapolation of ADC values for higher *b*-values, which cannot be obtained by the computed DWI method. However, this technique may not reduce the overall scan time; as in our scanning protocol, the scanning time to get three different *b*-values (*b*-400, 700, and 1000) is 1 min 39 sec and scanning time for one high *b*-value (*b*-2000) is 1 min 5 sec. This technique provides a method to obtain DW images and ADC values for a wide range of *b*-values.

According to the diffusion equation, *b*-value has a [time]^3^ dependency; thus, a very high *b*-value can be achieved in a clinical scanner with a moderate increase in the echo time (TE). However, the signal loss due to diffusion is a limiting factor at high *b*-values. The initial signal-to-noise ratio (SNR) and the tissue diffusion determine how quickly the signal goes below the noise level. As the tissue diffusivity is higher in normal tissue as compared to cancer tissue, normal region signal decay reaches to the noise level at a relatively faster rate. Hence, the observed signal at high *b*-values is dominated by the noise and appears to decay at a slower rate. This explains the reason of significant difference between ADC and sADC values at normal regions. As DWI signal attenuation is exponentially dependent on ADC, small changes in ADC can make a significant change in DWI contrast; this results in the significant increase of CR in sDWI images as compared to DWI.

The present study demonstrates that, although the higher *b*-value sDWI increases the contrast between lesion and normal tissue, the sADC shows similar contrast for *b*-1000, *b*-1500, and *b*-2000. This could be due to small cohort size of the patient with different Gleason scores, consistent with results in other studies [12, 24]. ADC computed from high *b*-value DWI has been shown to be more accurate in distinguishing prostate lesions from benign and normal tissues [25, 26]. Further investigation could be done for the clinical application of sDWI with larger patient populations. One of the limitations of our study was MRI examinations were not compared with a radical prostatectomy specimen. However, image-guided MR-overlayed biopsy could be a good alternative to radical prostatectomy where patient refuses to undergo prostatectomy.

Our initial investigation suggests that the ADC values corresponding to higher *b*-value DWI can be computed using a log-linear relationship derived from lower *b*-values (*b* ≤ 1000). Moreover, this computational method can also be manipulated to determine optimized *b*-values to create ADC maps. The synthetic ADC technique could be a useful tool to provide optimized image contrast for quantitative DW-MR imaging applications in oncology where ADC is routinely used in clinical practice.

## Figures and Tables

**Table 1:** Comparison between sADC values in lesions with Gleason score (GS) <6 and GS ≥6 at *b*-1000, *b*-1500, and *b*-2000.

	Malignant GS (6,7) *N* = 7 sADC (10^−3^ mm^2^/s) (mean ± SD)	Benign GS < 6 *N* = 8 sADC (10^−3 ^mm^2^/s) (mean ± SD)	95% CI	*p* value
*b*-1000	0.818 ± 0.067	1.131 ± 0.084	0.075–0.550	0.0138^*∗*^
*b*-1500	0.682 ± 0.059	1.007 ± 0.090	0.084–0.567	0.0121^*∗*^
*b*-2000	0.601 ± 0.057	0.935 ± 0.094	0.088–0.581	0.0116^*∗*^

^*∗*^Statistically significant. 95% CI, 95% confidence interval; sADC, synthetic apparent diffusion coefficient; GS, Gleason score.

## Data Availability

The data that support the findings of this study are available from the corresponding author upon reasonable request.

## References

[1] Vargas H. A., Akin O., Franiel T. (2011). Diffusion-weighted endorectal MR imaging at 3 T for prostate cancer: tumor detection and assessment of aggressiveness. *Radiology*.

[2] Tamada T., Prabhu V., Li J., Babb J. S., Taneja S. S., Rosenkrantz A. B. (2017). Assessment of prostate cancer aggressiveness using apparent diffusion coefficient values: impact of patient race and age. *Abdominal Radiology*.

[3] Kim T. H., Kim C. K., Park B. K. (2016). Relationship between Gleason score and apparent diffusion coefficients of diffusion-weighted magnetic resonance imaging in prostate cancer patients. *Canadian Urological Association Journal*.

[4] Lebovici A., Sfrangeu S. A., Feier D. (2014). Evaluation of the normal-to-diseased apparent diffusion coefficient ratio as an indicator of prostate cancer aggressiveness. *BMC Medical Imaging*.

[5] Quentin M., Blondin D., Klasen J. (2012). Comparison of different mathematical models of diffusion-weighted prostate MR imaging. *Magnetic Resonance Imaging*.

[6] Bennett K. M., Schmainda K. M., Bennett (Tong) R., Rowe D. B., Lu H., Hyde J. S. (2003). Characterization of continuously distributed cortical water diffusion rates with a stretched-exponential model. *Magnetic Resonance in Medicine*.

[7] Jafar M. M., Parsai A., Miquel M. E. (2016). Diffusion-weighted magnetic resonance imaging in cancer: reported apparent diffusion coefficients, in-vitro and in-vivo reproducibility. *World Journal of Radiology*.

[8] Kyo Kim C., Kwan Park B., Kim B., Kim C. K. (2010). High-b-value diffusion-weighted imaging at 3 T to detect prostate cancer: comparisons between b values of 1,000 and 2,000 s/mm^2^. *American Journal of Roentgenology*.

[9] de Perrot T., Scheffler M., Boto J. (2016). Diffusion in prostate cancer detection on a 3T scanner: how many b-values are needed?. *Journal of Magnetic Resonance Imaging*.

[10] Manenti G., Nezzo M., Chegai F., Vasili E., Bonanno E., Simonetti G. (2014). DWI of prostate cancer: optimal b-value in clinical practice. *Prostate Cancer*.

[11] Saritas E. U., Lee J. H., Nishimura D. G. (2011). SNR dependence of optimal parameters for apparent diffusion coefficient measurements. *IEEE Transactions on Medical Imaging*.

[12] Tamada T., Kanomata N., Sone T. (2014). High b value (2,000 s/mm^2^) diffusion-weighted magnetic resonance imaging in prostate cancer at 3 tesla: comparison with 1,000 s/mm^2^ for tumor conspicuity and discrimination of aggressiveness. *PLoS One*.

[13] Katahira K., Takahara T., Kwee T. C. (2011). Ultra-high-b-value diffusion-weighted MR imaging for the detection of prostate cancer: evaluation in 201 cases with histopathological correlation. *European Radiology*.

[14] Ueno Y., Takahashi S., Kitajima K. (2013). Computed diffusion-weighted imaging using 3-T magnetic resonance imaging for prostate cancer diagnosis. *European Radiology*.

[15] Blackledge M. D., Leach M. O., Collins D. J., Koh D.-M. (2011). Computed diffusion-weighted MR imaging may improve tumor detection. *Radiology*.

[16] Takeuchi M., Matsuzaki K., Harada M. (2016). Computed diffusion-weighted imaging for differentiating decidualized endometrioma from ovarian cancer. *European Journal of Radiology*.

[17] Ueno Y., Takahashi S., Ohno Y. (2015). Computed diffusion-weighted MRI for prostate cancer detection: the influence of the combinations of b-values. *The British Journal of Radiology*.

[18] Yoshida R., Yoshizako T., Katsube T., Tamaki Y., Ishikawa N., Kitagaki H. (2017). Computed diffusion-weighted imaging using 1.5-T magnetic resonance imaging for prostate cancer diagnosis. *Clinical Imaging*.

[19] Thörmer G., Otto J., Reiss-Zimmermann M. (2012). Diagnostic value of ADC in patients with prostate cancer: influence of the choice of b values. *European Radiology*.

[20] Peng Y., Jiang Y., Antic T. (2014). Apparent diffusion coefficient for prostate cancer imaging: impact of b values. *American Journal of Roentgenology*.

[21] Oto Y., Afaq A., Rowe D. B., Lu Y., Shukla-Dave A., Grover J. (2012). Diffusion-weighted magnetic resonance imaging of the prostate: improved robustness with stretched exponential modeling. *Journal of Computer Assisted Tomography*.

[22] Iima M., Le Bihan D. (2016). Clinical intravoxel incoherent motion and diffusion MR imaging: past, present, and future. *Radiology*.

[23] Understanding Your Pathology Report: Prostate Cancer

[24] Rosenkrantz A. B., Hindman N., Lim R. P. (2013). Diffusion-weighted imaging of the prostate: comparison of b1000 and b2000 image sets for index lesion detection. *Journal of Magnetic Resonance Imaging*.

[25] Taneja K., Kaji Y., Kuroda K., Sugimura K. (2008). High b-value diffusion-weighted imaging in normal and malignant peripheral zone tissue of the prostate: effect of signal-to-noise ratio. *Magnetic Resonance in Medical Sciences*.

[26] Kitajima K., Takahashi S., Ueno Y. (2012). Clinical utility of apparent diffusion coefficient values obtained using high b-value when diagnosing prostate cancer using 3 tesla MRI: comparison between ultra-high b-value (2000 s/mm^2^) and standard high b-value (1000 s/mm^2^). *Journal of Magnetic Resonance Imaging*.

